# Vine Waste Valorisation: Integrated Approach for the Prospection of Bioactive Lipophilic Phytochemicals

**DOI:** 10.3390/ijms20174239

**Published:** 2019-08-30

**Authors:** Ângelo C. Salvador, Mário M. Q. Simões, Artur M. S. Silva, Sónia A. O. Santos, Sílvia M. Rocha, Armando J. D. Silvestre

**Affiliations:** 1 QOPNA/LAQV-REQUIMTE, Department of Chemistry, University of Aveiro, Campus Universitário de Santiago, 3810-193 Aveiro, Portugal; 2 CICECO-Aveiro Institute of Materials, Department of Chemistry, University of Aveiro, Campus Universitário de Santiago, 3810-193 Aveiro, Portugal

**Keywords:** vine wastes, *Vitis vinifera* L. lipophilic compounds, bioactive phytochemicals, GC–MS, triterpenic compounds

## Abstract

Substantial amounts of vine wastes are produced during vineyard management, and the chemical profiling of high-value lipophilic phytochemicals is becoming crucial in order to find a complementary route towards their integrated valorisation. The prospection of bioactive phytochemicals from unripe grape, vine shoot, vine cane, stalk and leaf dichloromethane extracts was carried out by gas chromatography–mass spectrometry (GC–MS), analysing samples from a mixture of four red *Vitis vinifera* L. varieties (Baga, Aragonez, Água Santa and Shiraz), collected at Bairrada Appellation, as a representative case study of typical multi-variety Portuguese vineyards. Vine wastes showed distinct amounts of lipophilic extract, ranging from 0.68% (vine canes) to 13.35% (vine leaves) at dry weight (dw). Thirty-three components were identified, including fatty acids and alcohols, sterols and triterpenoids accounting for amounts from 118.9 mg/100 g dw to 1512.0 mg/100 g dw. The integrated study revealed that unripe grape, stalk and leaf dichloromethane extracts stood out as possible sources of triterpenic compounds (103.2 to 653.5 mg/100 g dw), with lupeol, ursolic and oleanolic acids prevailing. Leaf extract is also reported as an undervalued source of α-tocopherol, as the major component detected in this matrix (300.5 mg/100 g dw). These exploratory results are a relevant contribution for the exploitation of undervalued vine residues as a source of health-promoting components with the potential to be used as supplements or nutraceutical ingredients.

## 1. Introduction

Agricultural practices and agro-industrial activities generate millions of tons of wastes, which can ultimately lead to serious environmental concerns. Finding ways to reduce the impact of waste or co-products, while adding value to these residues and to the entire value chain, has become an important field of research [1]. The exploitation of crop wastes in particular, as a source of health-promoting ingredients, is an attractive opportunity not only to minimize their environmental impact but also for higher profitability [2]. A wide range of industries can consume these ingredients, including the food industry, in the formulation of supplements or as bioactive ingredients in cosmetic, pharmaceutical and nutraceutical industries [2].

The production of grape crops is one of the main agro-economic activities in the world, with up to 77 million tons produced every year [3], being consumed as fresh grapes or in the formulation of products, including wine (75–80% of grape production), jam, juice, jelly, raisins, vinegar and seed oil [4,5].

Viticulture has experienced an impressive global growth in acreage and value; however, substantial amounts of organic and inorganic waste are generated throughout the value chain, representing remarkable ecologic and economic waste management issues and challenges [5]. Currently, the concept of sustainable viticulture requires that the sector optimizes the available resources to improve environmentally responsible viticulture [6], emphasizing the urgent need to move towards more sustainable grape culture and wine production practices [5]. Nonetheless, viticulture still generates about 5 tons of solid waste per hectare annually during cultivation and harvesting [7]. Vineyard management often implies cane and vine-shoot pruning, leaf trimming and cluster thinning, mainly to address wine industry requirements, aiming to obtain wines with better sensorial quality. The generated waste includes grape stalks, unripe grapes, vine shoots, vine canes and leaves. Nonetheless, grape pomace is the main solid organic waste from winery industries and is the reason why most of the research is focused on this matrix [5], with the wastes mentioned above remaining largely unexploited. In fact, few efforts have been made to find ways to add value to these wastes, which can be an endless source of bioactive phytochemicals [8]. In addition, most of the research dealing with wastes from viticulture and wine-making processes has been focused on phenolic compounds (nearly 40% of the literature reports [9]), although other relevant families of compounds—e.g. sterols or triterpenes—also have remarkable bioactivities, and thus contribute to their valorisation. The presence of some of these compounds has been already reported in *V. vinifera* morphological parts, such as grapes [10] and stalks [11], although an integrated characterization of their lipophilic fraction including different families of compounds (i.e., fatty acids, long-chain aliphatic alcohols, sterols or triterpenes) is not available in the literature.

In this vein, a detailed chemical characterization of the lipophilic components of viticulture wastes is reported here, using samples composed of a mixture of four red varieties (*Vitis vinifera* L. Baga, Aragonez, Água Santa and Shiraz) collected at Bairrada Appellation as a representative case study of typical multi-variety Portuguese vineyards, which represents a step of paramount importance in promoting the valorization of natural products.

## 2. Results and Discussions

### 2.1. Chemical Composition of Vine Waste Lipophilic Extracts

The water content of the *V. vinifera* vine wastes ranged from 12.4% (vine canes) to 89.4% (unripe grapes) of the fresh samples’ weight. The five *V. vinifera* residues presented distinct (*p* < 0.05) dichloromethane (DCM) extract yields, with the vine leaves showing the highest yield (13.35%), followed by unripe grapes (2.81%), vine shoots (1.13%), grape stalks (0.72%) and finally vine canes (0.68%). With the exception of vine leaves, the extraction yields obtained are in the same range as those described in the literature for DCM extracts from lignocellulosic materials (usually up to 2%) [11,12,13]. Leaf DCM extracts from other species were also shown to reach relatively high extraction yields; for instance, the yield of DCM extracts from *Cynara cardunculus* L. var. *altilis* leaves was reported as 17.3% [14]. To the best of our knowledge, the studied red varieties (Baga, Aragonez, Água Santa and Shiraz) have not been characterised before in terms of lipophilic components. Thus, the results presented here are discussed in comparison to other red grape varieties available in the literature. 

It is important to note that several intrinsic and extrinsic factors affect the chemical composition of these matrices, and therefore their extraction yields. For instance, studies devoted to the chemical profiling of specific organs of different grape varieties reported that phenolic, fatty acid, tocopherol and sterol compositions are affected by the studied variety [15,16].

**Figure 1:**
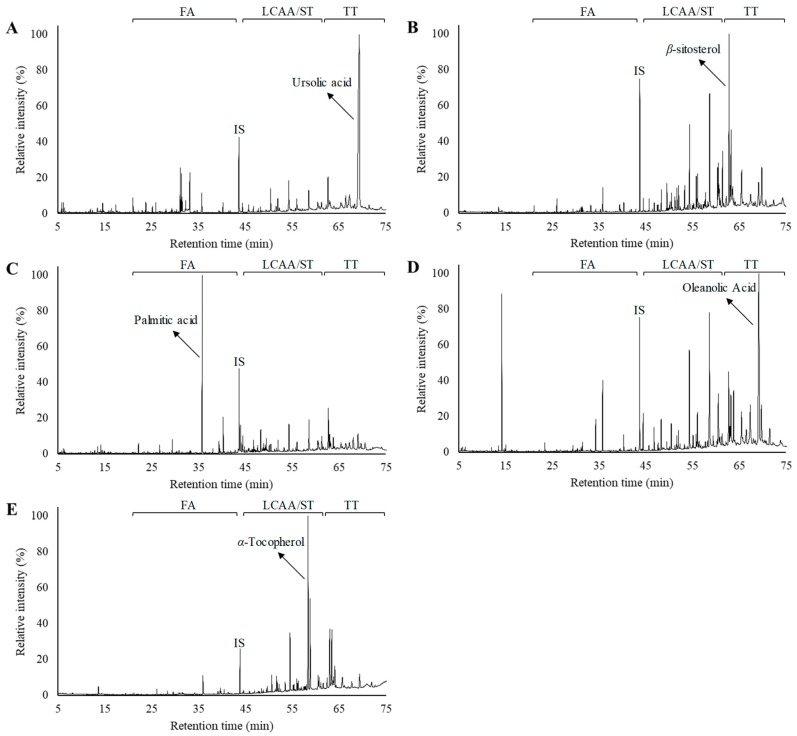
Gas chromatography–mass spectrometry (GC–MS) chromatograms of the derivatized lipophilic extracts from *V. vinifera* vine wastes: (**A**) unripe grapes, (**B**) vine shoots, (**C**) vine canes, (**D**) grape stalks, and (**E**) leaves. FA—fatty acids, LCAA—long chain aliphatic alcohols, ST—sterols, TT—triterpenoids, IS—internal standard. For each chromatogram, the major component was highlighted.

**Figure 2:**
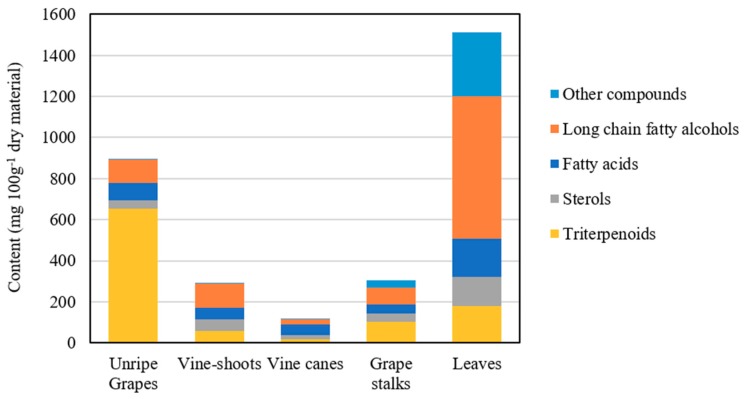
Major families of the lipophilic components identified in the *V. vinifera* unripe grape, vine shoot, vine cane, grape stalk and leaf extracts (mg/100 g of dry material).

The chemical composition of the DCM extracts of the residues, from the different morphological parts of *V. vinifera*, was studied in detail by GC–MS. A representative chromatogram of each derivatized lipophilic extract is shown in Figure 1. The main chemical families and major components are also indicated in Figure 1. The identification of the main lipophilic extracts and the corresponding quantification is summarised in Table 1, mainly composed by free saturated and unsaturated fatty acids (C_10_–C_30_), long-chain aliphatic alcohols (C_22_–C_30_), sterols, and triterpenic compounds, among others. The contents of the main chemical families are shown in Figure 2.

### 2.2. Fatty Acids

Free fatty acids accounted for 42.5 mg/100 g dw (grape stalks) to 182.8 mg/100g dw (grape leaves), ranging from 10–47% of the total identified lipophilic compounds (Figure 2). Palmitic acid was the main fatty acid detected in vine cane (shown in Figure 1C), stalk and leaf DCM extracts. Grape stalks, one of the most studied morphological part of grapes (in terms of the chemical characterization of DCM extracts), were previously reported as having considerable amounts of saturated and unsaturated fatty acids (up to 48%) [11], containing C_12_ to C_28_ skeletons [12]. Free fatty acids were also already reported in vine leaves with chain lengths ranging from C_10_ to C_24_, with palmitic acid likewise being one of the most abundant fatty acids [17,18]. Vine shoot lipophilic extract from the “Sauvignon” variety revealed that fatty acids represented 30.9% of the extract [19], with the major components being oleic, linoleic, palmitic, linolenic, stearic and behenic acids. The present study reports considerably lower fatty acid contents (19.0%) than those reported in previous studies, which, besides the analysed variety, could also be due to the harvest season and the edaphoclimatic and extraction conditions.

Saturated fatty acids stood out compared to unsaturated ones both in terms of the number of identified components—11 vs. 4, respectively—and in quantity—40.9–168.5 mg/100 g dw vs. 1.6–14.3 mg/100 g dw, respectively. Triacontanoic acid was the predominant fatty acid in unripe grapes, vine shoots, grape stalks and leaves, ranging from 9.9 (stalks) to 50.8 (leaves) mg/100 g dw. Although the information of the fatty acid profile in *V. vinifera* unripe grapes is scarce, structures from C_14_ to C_18_ have been reported, as well as a higher content of unsaturated fatty acids than saturated ones [19], in opposition to the observations in the present study. Considerable amounts of palmitic acid were detected, ranging from 6.0 mg/100 g dw in vine shoots to 45.5 mg/100 g dw in leaves.

Unsaturated fatty acids were present in relatively lower amounts than saturated ones, representing up to 8% of the overall fatty acid content. It is worth mentioning that leaves had a 6 to 9-fold higher content of unsaturated fatty acids (14.20 mg/100 g dw) than the other analysed vine residues. The occurrence of unsaturated fatty acids, and particularly the polyunsaturated fatty acids (PUFAs) ω-6 linoleic acid and ω-3 linolenic acid, is of major importance in the human diet as they have an important role in the prevention or even management of chronic diseases [20]. This illustrates the potential of vine leaves (due to the relatively high content compared to other residues) to be used as ingredients in food formulations or supplements that can be included in the human diet, which might contribute to the diary intake of these compounds.

### 2.3. Long-Chain Aliphatic Alcohols

Long-chain aliphatic alcohols were detected in all *V. vinifera* residues, with chain lengths ranging from 22 to 30-carbon. To the best of our knowledge, the presence of these components in unripe grapes, vine-shoots, and vine-canes is reported here for the first time. Vine leaves residues presented the highest amount of aliphatic alcohols, accounting for 693.4 mg/100 g dw (45.9% of the identified components), with octacosanol and triacontanol as the major components. The prevalence of this chemical family in *V. vinifera* leaves, representing up to 61% of the extract, has been reported in the literature, with hexacosanol, octacosanol and triacontanol being described as the major components [21,22]. This family also prevailed in stalks and vine shoots, representing, 27.6% and 40.5%, respectively, of the total identified compounds (Figure 2). Long-chain aliphatic alcohols (with an even number of carbon atoms between C_20_ to C_32_) were also reported on grape stalks extracts [11,12], representing up to 29% of the extract [11], similarly to the values presented here (27.6%). Long-chain aliphatic alcohols represented about 12.7% of the total identified components in unripe grapes, with a content of 114.3 mg/100 g dw. Although aliphatic alcohols were not reported before in unripe grapes, their presence in fresh and dried *V. vinifera* mature grapes—and in particular the high abundance of hexacosanol—has been described from a qualitative perspective [23]. Notwithstanding, the overall content of aliphatic alcohols has been described to be higher in seeds from unripe grapes compared to ripe ones [24], corroborating the idea that they are present in a relatively higher amount in unripe grapes when compared to ripe ones.

The health benefits of long-chain aliphatic alcohols have been already recognized. The intake of 10 to 20 mg per day of a mixture of these components (with octacosanol as major compound, 63%) lowered total and low-density lipoprotein (LDL) cholesterol and raised high-density lipoprotein (HDL) cholesterol levels [25]. Thus, the intake of a formulation containing ca. 1–3 g of dry *V. vinifera* leaf lipophilic extract potentially represents an active dosage intake, assuming the referred value (10–20 mg), which illustrates the nutraceutical potential of vine residues extracts.

### 2.4. Sterols

Sterols, which represented from 4.4% (unripe grapes) to 19.8% (vine-shoots) of the total identified lipophilic components, are a well-known family of bioactive components with remarkable value for several industries as dietary supplements and functional foods [26]. To the best of our knowledge, sterols are reported here for the first time as constituents of vine canes, although their abundance in other *V. vinifera* morphological parts, such as grapes [27], leaves [28], stalks [12] and vine shoots [19], has been already described. Four sterols, namely campesterol, stigmasterol, β-sitosterol and stigmastanol, were identified in vine shoots, vine canes and grape stalks, while only stigmasterol, β-sitosterol and stigmastanol were detected in unripe grapes. Campesterol and stigmastanol are reported for the first time as constituents of stalks and of both stalks and unripe grapes, respectively. A single sterol, namely β-sitosterol, was detected in vine leaves, in agreement with previously published data [28].

The total sterol content varied by ca. 10-fold between the different matrices; specifically, they ranged from 16.9 mg/100 g dw (vine canes) to 143.6 mg/100 g dw (leaves). β-Sitosterol was the most abundant sterol, except for grape stalks, which presented a higher content of stigmastanol. Actually, β-sitosterol was one of the major components present in vine shoot lipophilic extract, accounting for 35.4 mg/100 g dw and representing 12% of all identified compounds (Figure 1B). This compound was previously reported as one of the main phytosterols in unripe grapes [27] and stalks [11,12].

According to the European Food Safety Authority (EFSA), a daily dietary consumption of 2–2.4 g of sterols is linked to a 9% decrease of low-density lipoprotein (LDL)-cholesterol [29]. Therefore, vine wastes may be exploited as a source of bioactive sterols that may be incorporated in food formulations or supplements, due to their known role as cholesterol-lowering agents, reducing the risk of coronary heart diseases.

### 2.5. Triterpenic Components

The lipophilic composition of vine wastes demonstrates that these might be a very promising source of triterpenic compounds. The triterpenic compounds content ranged from 19.4 mg/100 g dw in vine canes to 653.5 mg/100 g dw in unripe grapes.

Ursolic acid was the only triterpenic compound detected in unripe grapes, accounting for up to 72.7% of the total identified compounds, as clearly seen in Figure 1A and Figure 2. The higher concentration of triterpenic components in unripe grapes, when compared to mature ones [21,22], highlights the interest of studying this waste as a source of bioactive components. The fact that other triterpenic components were reported in *V. vinifera* grape cuticular wax extracts [10,21,22] could be related to several factors such as the ripening stage, grape variety or edaphoclimatic conditions. Ursolic acid was also the major triterpenic component detected in vine shoot extracts (24.9 mg/100 g dw), while oleanolic acid was the major component for cane (7.5 mg/100 g dw) and stalk extracts (72.5 mg/100 g dw). This compound was also the major component detected in stalk extracts, corresponding to 23.8% of the total identified compounds, as shown in Figure 1D.

Triterpenic compounds were previously described as constituents of stalks; however, only betulin and β-amyrin were reported, representing only 2.26% of the total components detected [11]. Lupeol was the major triterpenic compound detected in leaves, accounting for 87.2 mg/100 g dw, followed by oleanolic acid (69.2 mg/100 g dw). As for grapes and stalks, a different triterpenic profile was previously reported for *V. vinifera* leaf extract, for which only the presence of squalene and α-amyrin was described [28]. Oleanolic acid was also described in the leaves of other *Vitis* species [22]. Minor amounts of lupeol were also found in vine canes and stalks, while α-amyrin was identified in vine shoot extracts. Despite the existence of some studies dealing with the valorisation of canes and vine shoot wastes as a source of phenolic compounds, such as *trans*-resveratrol and *trans*-viniferin [30], the extraction and characterization of the triterpenic components from these matrices has not previously been described elsewhere.

Ursolic and oleanolic acids are common triterpenic compounds in plants and are connected with a vast range of biological activities, and they are often used in cosmetics and nutraceutical formulations [31]. For instance, elderberry extracts rich in ursolic and oleanolic acids were reported to lower insulin secretion and resistance in *in vivo* models, illustrating their antidiabetic potential [32], thus representing a possible route for the valorisation of vine wastes.

### 2.6. Other Components

α-Tocopherol, the most bioactive form of vitamin E, was the major component detected in *V. vinifera* DCM leaf extract (Figure 1E), accounting for 300.5 mg/100 g dw. This component was previously reported in this matrix, being suggested as biomarker for the fungal resistance of *V. vinifera* leaves [28]. α-tocopherol was observed in grape stalk extract, accounting for 1.5 mg/100 g dw, a value similar to those previously published for the stalks of several *V. vinifera* varieties, including red grape varieties [33]. *α*-tocopherol is part of the antioxidant defence system and is a peroxyl radical scavenger, protecting PUFAs in particular within membrane phospholipids and plasma lipoproteins [34]. According to EFSA, the adequate intake of α-tocopherol is set between 5 and 13 mg/day depending on gender and age [34]. Therefore, an amount of 4.3 g of *V. vinifera* dry leaves (i.e., 577.6 mg of extract) attains the upper value of the daily reference value, highlighting the potential of this vine residue. Likewise, phytol was only detected in leaf extract (5.2 mg/100 g dw). This diterpene is a chlorophyll degradation product which is widely distributed in nature, with a broad range of bioactivities, and is already used as a fragrance ingredient and as a serious new drug candidate for pharmaceutical applications [35].

Other compounds, namely glycerol and azelaic acid, were found in all *V. vinifera* DCM extracts in a range between 0.4 (vine shoots) to 31.7 (grape stalks) mg/100 g dw and 0.7 (vine shoots and grape stalks) to 4.7 (leaves) mg/100 g dw, respectively. Finally, the monoglyceride 1-monopalmitin was observed in unripe grape, cane and stalk extracts presenting similar contents; i.e., from 0.7 to 0.8 mg/100 g dw. Glycerol and 1-monopalmitin were previously described in grape stalks [11,12].

## 3. Materials and Methods

### 3.1. Sampling and Handling

The studied samples belong to a mixture of four red *Vitis vinifera* varieties (Baga, Aragonez, Água Santa and Shiraz) collected in the Bairrada Appellation, Portugal, from the 2017 harvest. Unripe grapes, vine shoots, vine canes and leaves were obtained during the vineyard management operations, and the grape stalks were obtained by removing mature grapes from the clusters. Grape stalks, unripe grapes, vine shoots and leaves were freeze-dried using VirTis BenchTop K equipment (SP Industries, Gardiner, NY, USA), milled and stored at room temperature. Vine canes were milled and stored at room temperature (no freeze-drying was performed due to the relatively low water content of this biomass, ca. 12%). Water content was determined for all samples at 105 °C for 8 h.

### 3.2. Characterization of the Lipophilic Compounds

The lipophilic fraction of the different matrices was Soxhlet extracted with ca. 150 mL of dichloromethane (DCM) (Sigma Chemical Co., Madrid, Spain) for 8 h, using 4–12 g of dry sample [36]. The solvent was evaporated to dryness, the extracts weighed, and the results expressed as percentage of dry weight (% dw). Dichloromethane was only used for analytical purposes, since it has been well described as a specific solvent for lipophilic component extraction [37].

Before GC–MS analysis, about 20 mg of each dry extract was converted into its trimethylsilyl (TMS) derivatives according to a previously optimized methodology [37]. The derivatized extracts were analysed by GC–MS adapted from a methodology described previously [36,38] on a GC–MS-QP2010 Ultra (Shimadzu, Kyoto, Japan), equipped with a DB–1 J&W capillary column (30 m × 0.32 mm inner diameter, 0.25 μm film thickness). The following chromatographic conditions were used: initial temperature, 80 °C for 5 min; temperature gradient, 4 °C/min; final temperature, 260 °C; temperature gradient, 2 °C/min; final temperature, 285 °C for 13 min; injector temperature, 250 °C; transfer-line temperature, 290 °C; split ratio, 1:50. Helium was used as the carrier gas.

Compounds were identified as TMS derivatives by comparing their mass spectra with the GC–MS spectral library (Wiley 275 and U.S. National Institute of Science and Technology (NIST14)), by their characteristic retention times under the same GC conditions, and by comparing their mass spectra fragmentation profiles with published data [14,36,37,38,39,40,41,42], or by the injection of standards (ursolic acid, stigmasterol, hexadecanoic acid and nonadecan-1-ol).

For semi-quantitative analysis, GC–MS was calibrated with pure reference compounds representative of the major lipophilic extractive families (ursolic acid, stigmasterol, hexadecanoic acid and nonadecan-1-ol relative to tetracosane (Sigma Chemical Co., Madrid, Spain)). The respective response factors were calculated as an average of three GC–MS replicates. Two extracts were prepared for each sample, which were injected in duplicate (*n* = 4).

## 4. Conclusions

Vineyard management encompasses several practices to assure the appropriate growing of grapes, improving their potential and maintaining vineyard resources. Cane and vine shoot pruning, leaf trimming and cluster thinning generate relevant amounts of wastes during the entire phenological development of the vine, which prompted an integrated study of their lipophilic composition aimed at their future valorisation.

The lipophilic extraction yields ranged between 0.68% to 13.35%, accounting for values from 118.9 (vine canes) to 1512.0 (leaves) mg/100 g dw. This study reports 33 lipophilic components, belonging to fatty acids, long-chain aliphatic alcohols, sterols and triterpenic compounds. Fatty acids represented 10–47% of the total identified lipophilic compounds, in which palmitic acid and octadec-9-enoic acid stood out as saturated and unsaturated fatty acids, respectively. Long-chain fatty alcohols, from C_22_ to C_30_, represented up to 61% of the total identified compounds of leaves, with hexacosanol, octacosanol and triacontanol as the major components of this family. Sterols, which represented up to 19.8% (vine-shoots) of the total identified components, had β-sitosterol as the most abundant component, except for grape stalks, which presented a higher content of stigmastanol. The contents of triterpenic compounds ranged from 19.4 mg/100 g dw in vine canes to 653.5 mg/100 g dw in unripe grapes. Ursolic acid, oleanolic acid and lupeol were the main detected triterpenic compounds. α-Tocopherol was the major component found in grapes leaf DCM extract (300.5 mg/100 g dw), which was also shown to be a promising source of long-chain aliphatic alcohols. 

The obtained results demonstrated that undervalued vine wastes are a reliable source of bioactive compounds with high potential for use as ingredients for supplements or nutraceuticals.

Finally, the implementation of more environmentally friendly and safe extraction processes to obtain these extracts will be essential. In this context and based on our previous experience, namely in the supercritical CO_2_ extraction applied to other agro-forest residue lipophilic extracts [43,44,45,46], this will be the technique of choice to boost the valorisation of these vine wastes.

## Figures and Tables

**Table 1:** Compounds detected in the lipophilic extracts from *V. vinifera* vine wastes expressed in mg/100 g of dry material.

R.t. (min)	Compound	Unripe Grapes	RSD (%)	Vine-Shoots	RSD (%)	Vine Canes	RSD (%)	Grapes Stalks	RSD (%)	Leaves	RSD (%)
	**Fatty Acids**	**84.9**		**54.9**		**55.4**		**42.5**		**182.8**	
	*Saturated*										
19.6	Decanoic acid (C10:0)	n.d.		n.d.		n.d.		n.d.		2.4	8.0
25.6	Dodecanoic acid (C12:0)	n.d.		0.2	9.7	0.2	8.8	n.d.		n.d.	
30.9	Myristic acid (C14:0)	0.8	18.1	0.6	5.2	0.4	9.3	0.2	26.2	3.6	9.2
35.9	Palmitic acid (C16:0)	15.5	10.7	6.0	4.3	34.8	10.7	9.5	11.2	45.5	19.9
38.2	Heptadecanoic acid (C17:0)	n.d.		n.d.		0.7	11.2	n.d.		n.d.	
40.4	Stearic acid (C18:0)	6.0	23.7	1.8	10.5	5.9	11.7	2.7	30.7	8.7	20.3
44.5	Eicosanoic acid (C20:0)	6.5	10.3	2.8	2.2	2.9	12.4	5.0	7.5	7.4	6.3
48.4	Behenic acid (C22:0)	4.3	28.4	4.1	2.9	3.9	9.9	3.9	15.6	8.9	9.7
52.1	Lignoceric acid (C24:0)	12.7	12.7	5.2	8.7	2.1	6.4	2.9	21.0	18.2	10.9
56.1	Hexacosanoic acid (C26:0)	16.0	25.5	9.4	20.8	2.1	5.7	6.8	10.0	23.1	4.8
65.6	Triacontanoic acid (C30:0)	21.0	20.2	22.7	2.0	n.d.		9.9	30.8	50.8	13.3
	*Unsaturated*										
39.5	Linoleic acid (C18:2) + linolenic acid (C18:3)	0.6	30.2	1.3	27.1	1.7	13.0	1.0	19.6	5.6	31.2
39.7	Octadec-9-enoic acid (C18:1) isomer	1.1	33.5	0.4	36.0	0.6	14.0	0.3	12.3	6.6	7.3
39.8	Octadec-9-enoic acid (C18:1) isomer	0.5	0.0	0.5	29.8	0.2	8.9	0.3	8.2	2.0	17.6
	**Long-chain fatty alcohols**	**114.3**		**117.1**		**23.1**		**84.1**		**693.4**	
46.9	Docosan-1-ol	5.2	4.1	0.7	17.3	2.5	17.8	3.2	10.6	5.7	6.6
50.5	Tetracosan-1-ol	22.9	4.7	4.3	7.7	1.5	9.0	5.3	20.7	35.2	4.1
54.4	Hexacosan-1-ol	42.3	7.1	27.3	4.1	7.0	4.0	19.2	5.2	160.4	5.6
58.8	Octacosan-1-ol	32.5	23.2	48.7	6.1	9.0	3.2	38.6	6.0	292.9	8.8
63.3	Triacontan-1-ol	11.4	25.6	36.1	3.0	3.2	19.8	17.7	8.3	199.3	8.2
	**Sterols**	**40.0**		**57.2**		**16.9**		**40.5**		**143.6**	
60.8	Campesterol	n.d.		3.9	20.2	1.4	9.1	3.6	35.6	n.d.	
61.5	Stigmasterol	7.0	13.5	12.4	17.7	2.9	3.7	3.5	27.9	n.d.	
62.9	β-sitosterol	21.1	29.1	35.4	21.5	7.2	3.1	13.0	14.1	143.6	1.7
63.2	Stigmastanol	11.9	18.9	5.4	7.1	5.4	15.4	20.4	20.5	n.d.	
	**Triterpenic compounds**	**653.5**		**58.8**		**19.4**		**103.2**		**179.5**	
63.7	α-Amyrin	n.d.		14.5	10.4	n.d.		n.d.		n.d.	
64.0	Lupeol	n.d.		n.d.		3.7	11.7	17.9	28.2	87.2	3.0
69.4	Oleanolic acid	n.d.		19.5	3.7	7.5	3.7	72.5	16.1	69.2	5.0
70.6	Ursolic acid	653.5	4.0	24.9	5.2	4.6	7.7	n.d.		n.d.	
	Unidentified triterpenoids	n.d.		n.d.		3.6	15.5	12.7	14.9	23.2	14.9
	**Other compounds**	**5.6**		**1.1**		**4.2**		**34.6**		**312.7**	
14.3	Glycerol	1.4	21.6	0.4	6.5	1.1	15.0	31.7	24.6	2.3	9.5
29.47	Azelaic acid	3.5	16.9	0.7	4.6	2.3	16.4	0.7	10.7	4.7	14.6
39.12	Phytol	n.d.		n.d.		n.d.		n.d.		5.2	7.7
47.69	1-monopalmitin	0.8	18.8	n.d.		0.8	12.2	0.7	6.6	n.d.	
58.19	α-tocopherol	n.d.		n.d.		n.d.		1.5	31.8	300.5	2.5
	**Total content**	**898.3**		**289.1**		**118.9**		**304.8**		**1512.0**	

RSD—relative standard deviation; n.d.—not detected.

## Abbreviations

GC–MS	Gas Chromatography–Mass Spectrometry
DCM	Dichloromethane
dw	Dry weight
RSD	Relative standard deviation
PUFA	Polyunsaturated fatty acids
LD	Linear dichroism
LDL	Low-density lipoprotein
EFSA	European Food Safety Authority

## References

[1] Deiana A.C., Sardella M.F., Silva H., Amaya A., Tancredi N. (2009). Use of grape stalk, a waste of the viticulture industry, to obtain activated carbon. J. Hazard. Mater..

[2] Sánchez-Gómez R., Sánchez-Vioque R., Santana-Méridas O., Martín-Bejerano M., Alonso G.L., Salinas M.R., Zalacain A. (2017). A potential use of vine-shoot wastes: The antioxidant, antifeedant and phytotoxic activities of their aqueous extracts. Ind. Crops Prod..

[3] FAOSTAT Food and Agriculture - Organization of the United Nations Food and Agriculture Data. http://www.fao.org/faostat/en/#data/QC.

[4] Zhu F., Du B., Zheng L., Li J. (2015). Advance on the bioactivity and potential applications of dietary fibre from grape pomace. Food Chem..

[5] Beres C., Costa G.N.S., Cabezudo I., da Silva-James N.K., Teles A.S.C., Cruz A.P.G., Mellinger-Silva C., Tonon R.V., Cabral L.M.C., Freitas S.P. (2017). Towards integral utilization of grape pomace from winemaking process: A review. Waste Manag..

[6] Daane K.M., Vincent C., Isaacs R., Ioriatti C. (2016). Entomological Opportunities and Challenges for Sustainable Viticulture in a Global Market. Annu. Rev. Entomol..

[7] Zacharof M.P. (2017). Grape winery waste as feedstock for bioconversions: applying the biorefinery concept. Waste Biomass Valorization.

[8] Teixeira A., Baenas N., Dominguez-perles R., Barros A., Rosa E. (2014). Natural bioactive compounds from winery by-products as health promoters: A Review. Int. J. Mol. Sci..

[9] Santana-Méridas O., González-Coloma A., Sánchez-Vioque R. (2012). Agricultural residues as a source of bioactive natural products. Phytochem. Rev..

[10] Pensec F., Pączkowski C., Grabarczyk M., Woźniak A., Bénard-Gellon M., Bertsch C., Chong J., Szakiel A. (2014). Changes in the triterpenoid content of cuticular waxes during fruit ripening of eight grape (*Vitis vinifera*) Cultivars Grown in the Upper Rhine Valley. J. Agric. Food Chem..

[11] Pujol D., Liu C., Fiol N., Olivella M.À., Gominho J., Villaescusa I., Pereira H. (2013). Chemical characterization of different granulometric fractions of grape stalks waste. Ind. Crops Prod..

[12] Ping L., Brosse N., Sannigrahi P., Ragauskas A. (2011). Evaluation of grape stalks as a bioresource. Ind. Crops Prod..

[13] Prozil S.O., Evtuguin D.V., Lopes L.P.C. (2012). Chemical composition of grape stalks of *Vitis vinifera* L. from red grape pomaces. Ind. Crops Prod..

[14] Ramos R.A., Guerra A.R., Guerreiro O., Freire C.S.R., Silva A.M.S., Duarte M.F., Silvestre A.J.D. (2013). Lipophilic extracts of *Cynara cardunculus* L. var. altilis (DC): A source of valuable bioactive terpenic compounds. J. Agric. Food Chem..

[15] Alvarruiz A., Pardo J.E., Fernández E., Rubio M., Alvarruiz A., Alonso G.L. (2016). Characterization of grape seed oil from different grape varieties (*Vitis Vinifera*). Eur. J. Lipid Sci. Technol..

[16] Fernandes L., Casal S., Cruz R., Alberto J., Ramalhosa E. (2013). Seed oils of ten traditional Portuguese grape varieties with interesting chemical and antioxidant properties. Food Res. Int. J..

[17] Hebash K.A.H., Fadel H.M., Soliman M.M. (1991). Volatile components of grape leaves. J. Islamic Acad. Sci..

[18] Miele A., Bouard J., Bertrand A. (1993). Fatty acids from lipid fractions of leaves and different tissues of cabernet sauvignon grapes. Am. J. Enol. Vitic..

[19] Pecher V., Andre P. (2015). Method for preparing a lipophilic vine extract.

[20] Simopoulos A.P. (2008). The importance of the Omega-6/Omega-3 fatty acid ratio in cardiovascular disease and other chronic diseases. Exp. Biol. Med..

[21] Radler F. (1965). The surface waxes of the sultana vine (*Vitis vinifera* cv. Thompson seedless). Aust. J. Biol. Sci..

[22] Radler F. (1965). The main constituents of the surface waxes of varieties and species of the genus Vitis. Am. J. Enol. Vitic..

[23] Radler F., Horn D. (1965). The composition of grape cuticle wax. Aust. J. Chem..

[24] Rubio M., Alvarez-Ortí M., Andrés A., Fernández E., Pardo J.E. (2009). Characterization of oil obtained from grape seeds collected during berry development. J. Agric. Food Chem..

[25] Gouni-Berthold I., Berthold H.K. (2002). Policosanol: Clinical pharmacology and therapeutic significance of a new lipid-lowering agent. Am. Heart J..

[26] Berger A., Jones P.J.H., Abumweis S.S. (2004). Plant sterols: Factors affecting their efficacy and safety as functional food ingredients. Lipids Health Dis..

[27] Ruggiero A., Vitalini S., Burlini N., Bernasconi S., Iriti M. (2013). Phytosterols in grapes and wine, and effects of agrochemicals on their levels. Food Chem..

[28] Batovska D.I., Todorova I.T., Nedelcheva D.V., Parushev S.P., Atanassov A.I., Hvarleva T.D., Djakova G.J., Bankova V.S., Popov S.S. (2008). Preliminary study on biomarkers for the fungal resistance in *Vitis vinifera* leaves. J. Plant Physiol..

[29] Løvik M., Marchelli R., Martin A., Moseley B., Van Den Berg H., Van Loveren H., Verhagen H. (2008). Plant sterols and blood cholesterol scientific substantiation of a health claim related to plant sterols and lower/reduced blood cholesterol and reduced risk of (coronary) heart disease. EFSA J..

[30] Rayne S., Karacabey E., Mazza G. (2008). Grape cane waste as a source of trans-resveratrol and trans-viniferin: High-value phytochemicals with medicinal and anti-phytopathogenic applications. Ind. Crops Prod..

[31] Domingues R.M., Guerra A.R., Duarte M., Freire C.S., Neto C.P., Silva C.M., Silvestre A.J. (2014). Bioactive triterpenic acids: From agroforestry biomass residues to promising therapeutic tools. Mini. Rev. Org. Chem..

[32] Salvador Â.C., Król E., Lemos V.C., Santos S.A.O., Bento F.P.M.S., Costa C.P., Almeida A., Szczepankiewicz D., Kulczyński B., Krejpcio Z. (2017). Effect of elderberry (*Sambucus nigra* L.) extract supplementation in STZ-induced diabetic rats fed with a high-fat diet. Int. J. Mol. Sci..

[33] Tangolar S.G., Özogul F., Tangolar S., Yağmur C. (2011). Tocopherol content in fifteen grape varieties obtained using a rapid HPLC method. J. Food Compos. Anal..

[34] (2015). EFSA Scientific opinion on dietary reference values for vitamin E as α-tocopherol. EFSA J..

[35] Islam T., Vinícius M., Barros O., Alencar D., Mendes R., Freitas D. (2015). Chemico-biological interactions phytol in a pharma-medico-stance. Chem. Biol. Interact..

[36] Salvador Â.C., Rocha S.M., Silvestre A.J.D. (2015). Lipophilic phytochemicals from elderberries (*Sambucus nigra* L.): Influence of ripening, cultivar and season. Ind. Crops Prod..

[37] Domingues R.M.A., Sousa G.D.A., Silva C.M., Freire C.S.R., Silvestre A.J.D., Neto C.P. (2011). High value triterpenic compounds from the outer barks of several *Eucalyptus* species cultivated in Brazil and in Portugal. Ind. Crops Prod..

[38] Fonseca D.F.S., Salvador Â.C., Santos S.A.O., Vilela C., Freire C.S.R., Silvestre A.J.D., Rocha S.M. (2015). Bioactive phytochemicals from wild *Arbutus unedo* L. Berries from different locations in portugal: Quantification of lipophilic components. Int. J. Mol. Sci..

[39] Villaverde J.J., Vega A., Ligero P., Freire C.S.R., Neto C.P., Silvestre A.J.D. (2010). *Miscanthus x giganteus* bark organosolv fractionation: fate of lipophilic components and formation of valuable phenolic by-products. J. Agric. Food Chem..

[40] Sousa A.F., Pinto P.C.R.O., Silvestre A.J.D., Neto C.P. (2006). Triterpenic and other lipophilic components from industrial cork by-products. J. Agric. Food Chem..

[41] Freire C.S.R., Silvestre A.J.D., Neto C.P. (2002). Identification of new hydroxy fatty acids and ferulic acid esters in the wood of Eucalyptus globulus. Holzforschung.

[42] Freire C.S.R., Silvestre A.J.D., Neto C.P., Cavaleiro J.A.S. (2002). Lipophilic extractives of the inner and outer bark of *Eucalyptus globulus*. Holzforschung.

[43] De Melo M.M.R., Şen A., Silvestre A.J.D., Pereira H., Silva C.M. (2017). Experimental and modeling study of supercritical CO_2_ extraction of Quercus cerris cork: Influence of ethanol and particle size on extraction kinetics and selectivity to friedelin. Sep. Purif. Technol..

[44] Domingues R.M.A., Oliveira E.L.G., Freire C.S.R., Couto R.M., Simoes P.C., Neto C.P., Silvestre A.J.D., Silva C.M. (2012). Supercritical fluid extraction of *Eucalyptus globulus* bark-A promising approach for triterpenoid production. Int. J. Mol. Sci..

[45] Domingues R.M.A., de Melo M.M.R., Neto C.P., Silvestre A.J.D., Silva C.M. (2012). Measurement and modeling of supercritical fluid extraction curves of *Eucalyptus globulus* bark: Influence of the operating conditions upon yields and extract composition. J. Supercrit. Fluids.

[46] De Melo M.M.R., Silva R.P., Silvestre A.J.D., Silva C.M. (2016). Valorization of water hyacinth through supercritical CO_2_ extraction of stigmasterol. Ind. Crops Prod..

